# Prediction of thrombo‐embolic risk in patients with hypertrophic cardiomyopathy (HCM Risk‐CVA)

**DOI:** 10.1002/ejhf.316

**Published:** 2015-07-16

**Authors:** Oliver P. Guttmann, Menelaos Pavlou, Constantinos O'Mahony, Lorenzo Monserrat, Aristides Anastasakis, Claudio Rapezzi, Elena Biagini, Juan Ramon Gimeno, Giuseppe Limongelli, Pablo Garcia‐Pavia, William J. McKenna, Rumana Z. Omar, Perry M. Elliott, for the Hypertrophic Cardiomyopathy Outcomes Investigators

**Affiliations:** ^1^ The Inherited Cardiac Diseases UnitThe Heart Hospital/University College LondonLondonUK; ^2^ Department of Statistical ScienceUniversity College LondonLondonUK; ^3^ Cardiology Department and Research UnitA Coruña University HospitalGalician Health ServiceSpain; ^4^ Unit of Inherited Cardiovascular Diseases, 1st Department of CardiologyUniversity of AthensAthensGreece; ^5^ Institute of Cardiology, Department of Specialised, Experimental and Diagnostic MedicineUniversity of BolognaBolognaItaly; ^6^ Cardiac DepartmentUniversity Hospital Virgen ArrixacaMurcia‐Cartagena s/n, El PalmarMurciaSpain; ^7^ Monaldi HospitalSecond University of NaplesNaplesItaly; ^8^ Heart Failure and Inherited Cardiac Diseases UnitHospital Universitario Puerta del Hierro MajadahondaMadridSpain; ^9^ Biostatistics Group, University College London Hospitals/University College London Clinical Research CentreUniversity College LondonLondonUK

**Keywords:** Hypertrophic cardiomyopathy, Atrial fibrillation, Thrombo‐embolism

## Abstract

**Aims:**

Atrial fibrillation (AF) and thrombo‐embolism (TE) are associated with reduced survival in hypertrophic cardiomyopathy (HCM), but the absolute risk of TE in patients with and without AF is unclear. The primary aim of this study was to derive and validate a model for estimating the risk of TE in HCM. Exploratory analyses were performed to determine predictors of TE, the performance of the CHA_2_DS_2_‐VASc score, and outcome with vitamin K antagonists (VKAs).

**Methods and results:**

A retrospective, longitudinal cohort of seven institutions was used to develop multivariable Cox regression models fitted with pre‐selected predictors. Bootstrapping was used for validation. Of 4821 HCM patients recruited between 1986 and 2008, 172 (3.6%) reached the primary endpoint of cerebrovascular accident (CVA), transient ischaemic attack (TIA), or systemic peripheral embolus within 10 years. A total of 27.5% of patients had a CHA_2_DS_2_‐VASc score of 0, of whom 9.8% developed TE during follow‐up. Cox regression revealed an association between TE and age, AF, the interaction between age and AF, TE prior to first evaluation, NYHA class, left atrial (LA) diameter, vascular disease, and maximal LV wall thickness. There was a curvilinear relationship between LA size and TE risk. The model predicted TE with a C‐index of 0.75 [95% confidence interval (CI) 0.70–0.80] and the D‐statistic was 1.30 (95% CI 1.05–1.56). VKA treatment was associated with a 54.8% (95% CI 31–97%, P = 0.037) relative risk reduction in HCM patients with AF.

**Conclusions:**

The study shows that the risk of TE in HCM patients can be identified using a small number of simple clinical features. LA size, in particular, should be monitored closely, and the assessment and treatment of conventional vascular risk factors should be routine practice in older patients. Exploratory analyses show for the first time evidence for a reduction of TE with VKA treatment. The CHA_2_DS_2_‐VASc score does not appear to correlate well with the clinical outcome in patients with HCM and should not be used to assess TE risk in this population.

## Introduction

Hypertrophic cardiomyopathy (HCM) is a myocardial disorder characterized by LV hypertrophy not explained solely by abnormal loading conditions. It is inherited as an autosomal dominant trait and caused by mutations in cardiac sarcomere protein genes.^1^, ^2^, ^3^ Atrial fibrillation (AF) and thrombo‐embolism (TE) are common complications of the disease and are associated with adverse clinical outcomes and reduced survival.^4^, ^5^, ^6^, ^7^, ^8^, ^9^ However, HCM is a heterogeneous disorder with very variable clinical presentation, and the absolute risk of TE—and by implication the likely benefit from treatment—in individual patients with different clinical characteristics is unknown.^1^, ^10^, ^11^, ^12^

The primary aim of this study was to derive and validate a risk model for estimating the risk of TE in patients with HCM. Exploratory analyses were performed to determine clinical predictors of TE, the performance of the CHA_2_DS_2_‐VASc score,^13^ and the outcome of therapy with vitamin K antagonists (VKAs) in HCM patients with AF.

## Methods

### Study design and overview

Data from a retrospective, multicentre longitudinal cohort—the Hypertrophic Cardiomyopathy Outcome Investigators (www.HCMRisk.org)^14^—were used in the development of the prognostic model.

The study conforms to the principles of the Helsinki declaration. The sponsors of this study did not have a role in study design, data collection, analysis, and interpretation. O.G., M.P., R.O., and P.E. had access to all data and final responsibility to submit the article. The authors from each centre guarantee the integrity of data from their institution. All investigators have agreed to the article as written.

### Study population and participating centres

The study cohort consisted of all consecutively evaluated patients with HCM, followed at seven European centres: (i) The Heart Hospital, London, UK; (ii) A Coruna University Hospital, A Coruna, Spain; (iii) Unit of Inherited Cardiovascular diseases, 1st Department of Cardiology, University of Athens, Greece; (iv) Institute of Cardiology, University of Bologna, Italy; (v) University Hospital Virgen de la Arrixaca, Murcia, Spain; (vi) Monaldi Hospital, Second University of Naples, Italy; and (vii) Hospital Universitario Puerta del Hierro, Madrid, Spain. Some patients from this cohort are reported in other recently published studies.^14^, ^15^, ^16^, ^17^, ^18^, ^19^, ^20^, ^21^, ^22^, ^23^, ^24^, ^25^

Only adult patients (≥16 years of age) were studied. HCM was defined as a maximum LV wall thickness ≥15 mm unexplained solely by loading conditions^1^ or in accordance with published criteria for the diagnosis of disease in relatives of patients with unequivocal disease.^26^ Patients with known inherited metabolic diseases or syndromic causes of HCM were excluded from the study. Patients with a history of AF that had experienced TE prior to first evaluation at the centre were also excluded from the analysis.

### Ethics approval

Patients at A Coruna University Hospital (Spain), 1st Department of Cardiology, University of Athens (Greece), University Hospital Virgen de la Arrixaca (Spain), and Monaldi Hospital (Italy) provided written informed consent. The data collection at The Heart Hospital (UK) and Hospital Universitario Puerta de Hierro (Spain) have been approved by the appropriate ethics committee. The ethics committee at the Institute of Cardiology at the University of Bologna (Italy) were informed, but approval was not required under local research governance arrangements.

### Patient assessment and data collection

Patients were reviewed every 6–12 months or earlier if there was a change in symptoms. All patients underwent clinical assessment, pedigree analysis, physical examination, resting and ambulatory ECG, and transthoracic echocardiography. Each centre collected data independently using the same methodology.

### Clinical outcomes

The primary outcome was a thrombo‐embolic event defined as a composite of cerebrovascular accident (CVA), transient ischaemic attack (TIA), or systemic peripheral embolus as defined in the CHA_2_DS_2_‐VASc score.^10^, ^12^ A CVA was defined as a focal neurological deficit of sudden onset as diagnosed by a neurologist, lasting >24 h and caused by ischaemia. A TIA was defined as a focal neurological deficit of sudden onset as diagnosed by a neurologist, lasting <24 h. Peripheral embolism was defined as TE outside the brain, heart, eyes, and lungs.^13^

### Selection of predictors and coding

Following a review of the literature completed in September 2012, predictors of TE that have been reported previously in patients with HCM were considered as candidate predictor variables.^27^ Clinical parameters were used as pre‐specified predictors only when associated with TE in at least one published study, and were uniformly defined in all centres. In addition, predictors included in the CHA_2_DS_2_‐VASc score, and maximal LV wall thickness and peak left ventricular outflow tract (LVOT) gradient were considered for the analysis (*Table* 1). Clinically plausible interactions between selected variables such as age and AF and age and vascular disease were also assessed. All predictors were assessed at baseline evaluation.

**Table 1:** Definition of pre‐specified predictor variables assessed at baseline evaluation

Predictor variable	Definition	Coding
Sex	Male or female^44^	Binary, male/female
Age	Age at first evaluation in participating centres^43^, ^45^	Continuous, years
VKA	Use of vitamin K antagonist at first evaluation	Binary, yes/no
AF	Detection of paroxysmal, permanent of persistent AF on ECG or Holter monitoring^43^, ^45^	Binary, yes/no
TE	Thrombo‐embolism: CVA, TIA, peripheral embolus, as per CHA_2_DS_2_‐VASc score^10^, ^12^	Binary, yes/no
NYHA	NYHA class at first evaluation^45^	Categorical, I, II and III–IV
LA	Anterior–posterior left atrial diameter determined by 2D echocardiography in the parasternal long axis–short axis plane at time of first evaluation^44^	Continuous, mm
MWT	The greatest LV wall thickness measured at the level of the mitral valve, papillary muscles, and apex in the parasternal short axis plane using 2D echocardiography at time of evaluation^46^	Continuous, mm
FS	LV end‐diastolic dimension – LV end‐systolic dimension)/LV end‐diastolic dimension measured by M‐Mode or 2D echocardiography at time of evaluation^44^	Continuous, %
LVOT max	The maximum LV outflow gradient determined at rest and with Valsalva provocation (irrespective of concurrent medical treatment) using pulsed and continuous wave Doppler from the apical three‐ and five‐chamber views Peak outflow tract gradients were determined using the modified Bernoulli equation: gradient = 4*V* 2, where *V* is the peak aortic outflow velocity^47^	Continuous, mmHg
Hypertension	Diagnosis of hypertension prior to first evaluation, as per CHA_2_DS_2_‐VASc score^10^, ^12^	Binary, yes/no
Diabetes	Diagnosis of diabetes prior to first evaluation, as per CHA_2_DS_2_‐VASc score^10^, ^12^	Binary, yes/no
Vascular disease	Myocardial infarction, complex aortic plaque, and peripheral arterial disease, as per CHA_2_DS_2_‐VASc score^10^, ^12^	Binary, yes/no
Heart failure	Heart failure, especially moderate to severe LV systolic dysfunction, defined arbitrarily as LVEF <40% (calculated by FS), as per CHA_2_DS_2_‐VASc score^10^, ^12^	Binary, yes/no

FS, fractional shortening; LA, left atrial size, LVOT max, maximum LV outflow gradient; MWT, aximal wall thickness; TE, thrombo‐embolic event; VKA, vitamin K antagonist.

### Sample size

A minimum of 10 thrombo‐embolic events were required per coefficient estimated by the model to ensure that the regression coefficients of the model were estimated with adequate precision.^28^ The 172 TE endpoints observed in this cohort over a 10‐year follow‐up period allow the estimation of up to 17 regression coefficients with adequate precision and were sufficient for development of the risk model.

### General statistical methods

STATA (version 12) and R (version 3.0) were used for the statistical analyses. For descriptive results, variables are expressed as the mean ± standard deviation (SD), median and interquartile range (IQR), or counts and percentages as appropriate. The follow‐up time for each patient was calculated from the date of their first evaluation at participating centres to the date of the study endpoint, death, heart transplantation, cerebral haemorrhage, or the date of their most recent evaluation. The annual event rate was calculated by dividing the number of patients reaching the endpoint by the total follow‐up period for that endpoint. The Kaplan–Meier method was used to estimate the cumulative probability for the occurrence of an outcome.

### Missing data

The characteristics of patients with missing information were compared with those of patients with complete information to investigate bias due to missing data. Logistic regression was used to identify the predictors of missingness. Data were assumed to be missing at random, and values for the missing predictors were imputed using multiple imputation techniques based on chained equations.^29^ The multiple imputation model included all predictors of missingness, the outcome, all pre‐specified predictors of the risk model, and the estimate of the cumulative hazard function.^30^ Rubin's rules were used to combine the estimates from 30 imputed data sets.^31^

### Model development

All pre‐specified predictors were candidates for inclusion in the final risk model. To account for potential non‐linear relationships, we considered the addition of quadratic terms for all continuous predictors. Due to sample size issues, this was done in a pre‐selection procedure where bi‐variable models for each predictor and its quadratic term were fitted. Suspected interactions were also examined in a similar manner.

The model was developed based on the 172 events within the first 10 years of follow‐up. Backward elimination with a 15% significance level was used to select the predictors for the final risk model.^32^ Centre was not included to allow the model to be used in patients from other centres. However, a sensitivity analysis for centre effect was performed by including centre in the model. The proportional hazards assumption required by the Cox model was investigated using Schoenfeld residuals.^33^ The risk model was developed using the entire cohort.

### Model validation

Bootstrapping was used to evaluate the performance of the model. This is the most efficient internal validation procedure as all aspects of the model development, including variable selection, are validated.^34^ Two hundred bootstrap samples were generated for each imputed data set, and the optimism‐adjusted performance measures from the imputed data sets were combined using Rubin's rules.^35^ The calibration slope was used to assess the degree of agreement between the observed and predicted hazards of TE.^36^ A value close to 1 suggests good overall agreement. Graphical comparisons of the observed and predicted TE at 5 years were performed. The C‐index and D‐statistic were used to measure how well the model discriminated between patients with high and low risk of TE.^14^, ^37^, ^38^, ^39^ A value of 0.5 for the C‐index indicates no discrimination, and a value equal to 1 indicates perfect discrimination. The D‐statistic can be interpreted as the log hazard ratio (HR) for having TE between the low and high risk groups of patients. A model with no discriminatory ability results in a value of 0 for the D‐statistic, with increasing values indicating greater separation.

### Model presentation

The probability of TE at 5 years for an individual patient was calculated using the following equation, derived from the Cox proportional hazards model:

$$P_{TE\;\mathrm{at}\;5\;\text{years}}=1–S_{0}{\left(t\right)}^{\exp\;(\text{prognostic index})}$$

where *S*_0_(*t*) is the average survival probability at time *t* (i.e. at 5 years), and the prognostic index is the sum of the products of the predictors and their coefficients.

### Calculation of the CHA_2_DS_2_‐VASc score

The CHA_2_DS_2_‐VASc score was calculated for a subset of patients with AF not treated with a VKA at baseline.^10^, ^12^, ^13^ The distribution of the score and number of events according to the score was determined.

### Clinical outcome of anticoagulation in patients with atrial fibrillation

The incidence of TE in patients with AF who were and were not treated with anticoagulants was investigated using an intention to treat analysis. If a patient received treatment with a VKA at any time prior to the event, it was assumed that they continued on this medication for the duration of the study. The numbers of patients with and without TE that did or did not receive a VKA were compared. The absolute risk reduction (ARR) and number needed to treat (NNT) were calculated for patients in AF at an exploratory threshold of 4% risk of a thrombo‐embolic event over 5 years.

### Relationship of patient characteristics to thrombo‐embolic risk

Pre‐specified subanalyses were performed to assess the relationship between the 5‐year risk of TE and age, vascular disease, and left atrial (LA) size. Secondary analyses were performed in patients in sinus rhythm (SR) who experienced TE and patients with AF who did not experience TE.

## Results

### Baseline clinical characteristics

During the study period, 5104 patients were evaluated, of whom 197 were seen only once for baseline evaluation and were excluded from the analysis. Eighty‐six patients with a history of AF and TE prior to first evaluation were excluded. The final study cohort consisted of the remaining 4821 patients, and the baseline clinical characteristics are shown in *Table* 2.

**Table 2:** Clinical characteristics at baseline of the whole cohort and in patients with and without a thrombo‐embolic endpoint

Predictor	Whole cohort		No TE		TE	
	Total	Mean (SD)/*n* (%)	Total	Mean (SD)/*n* (%)	Total	Mean (SD)/*n* (%)
Age	4817	48.99 (16.40)	4645	48.74 (16.39)	172	55.73 (15.40)
LA	4627	43.97 (7.74)	4460	43.82 (7.68)	167	47.83 (8.37)
MWT	4768	19.44 (5.15)	4599	19.42 (5.18)	169	20.05 (4.16)
FS	4358	0.41 (0.10)	4198	0.41 (0.10)	160	0.40 (0.09)
LVOT max	4168	31.95 (40.94)	4023	31.85 (40.96)	145	34.72 (40.46)
Female	4820	1740 (36.10)	4648	1666 (35.84)	172	74 (43.02)
Prior TE	4821	80 (1.66)	4649	71 (1.53)	172	9 (5.23)
AF	4815	600 (12.46)	4643	552 (11.89)	172	48 (27.91)
VKA	4818	443 (9.20)	4646	410 (8.82)	172	33 (19.19)
NYHA II	4615	1584 (34.32)	4450	1519 (34.13)	165	65 (39.29)
NYHA III, IV	4615	494 (10.70)	4450	456 (10.24)	165	38 (23.03)
Vascular disease	3588	89 (2.48)	3438	79 (22.98)	150	10 (6.67)
Hypertension	4712	1414 (30.00)	4541	1354 (29.82)	171	60 (35.09)
Diabetes	4020	293 (7.29)	3868	279 (7.21)	152	14 (9.21)

Breakdown of AF in the whole cohort: paroxysmal 314, persistent 102, permanent 181, not specified 3.

FS, fractional shortening; LA, left atrial size, LVOT max, maximum LV outflow gradient; MWT, aximal wall thickness; SD, standard deviation; TE, thrombo‐embolic event; VKA, vitamin K antagonist.

### Thrombo‐embolic events during follow‐up

During a follow‐up period of 28 330.6 patient‐years (median 6.0 years, IQR = 3–9.7), 172 (3.6%) patients reached the primary endpoint within 10 years from first evaluation (105 CVA, 53 TIA, and 14 peripheral emboli); and 107 (2.2%) patients within the first 5 years. The 5‐ and 10‐year cumulative incidences were 2.9% [95% confidence interval (CI) 2.37–3.48%) and 6.4% (95% CI 5.42–7.53%), respectively. The clinical characteristics of patients with and without TE are shown in *Table* 2.

Patients in SR at first evaluation who developed TE during follow‐up were older (55.0 years vs. 47.5 years; difference in means = 7.5 years; 95% CI 4.60–10.42), had larger LA diameter (46.0 mm vs. 43.0 mm; difference in means = 3.0 mm; 95% CI 1.7–4.32), and were more symptomatic (NYHA III, IV) (14.4% vs. 9.0%; difference in proportions = 0.054; 95% CI 0.0099–0.1181) compared with patients who did not have an event. There was also a higher percentage of patients with vascular disease (5.7% vs. 2.0%; difference in proportions = 0.037; 95% CI 0.0074–0.0812) in the event cohort (Supplementary material online, *Table S1*). The mean age of patients with and without vascular disease was 62.4 and 49.1 years, respectively.

### Missing data

Missing data per variable are described in the Supplementary material online, *Table S2*.

### Model development

Univariable analyses are shown in *Table* 3. Only maximal LV wall thickness was found to have a non‐linear association with TE, and so a quadratic term was included as a candidate for the final prognostic model. The interaction between AF and age was also found to be significant. There were 15 predictors (16 regression coefficients) which were candidates for the final model.

**Table 3:** Exploratory univariable and multivariable analysis for predictors of thrombo‐embolism in hypertrophic cardiomyopathy

Univariable analysis				Multivariable analysis			
Predictor	HR	*P*‐value	95% CI	Predictor	HR	*P*‐value	95% CI
Sex	1.43	0.02	1.06–1.93	AGE	1.03	<0.001	1.02–1.04
AGE10	1.45	<0.001	1.31–1.60	AF	8.41	<0.001	1.95–36.35
AF	3	<0.001	2.15–4.19	age_af	0.97	0.03	0.95–1.00
Prior TE	4.15	<0.001	2.12–8.13	Prior TE	3.63	<0.001	1.81–7.29
NYHA II	1.61	0.01	1.14–2.29	NYHA II	1.25	0.21	0.88–1.78
NYHA III, IV	3.66	<0.001	2.44–5.48	NYHA III, IV	2.07	<0.001	1.35–3.17
LA5	1.36	<0.001	1.24–1.48	LA	1.03	<0.001	1.01–1.05
MWT	1.01	0.3	0.99–1.04	MWT	1.45	<0.001	1.12–1.88
FS	0.22	0.08	0.04–1.20	MWT^2^	0.99	0.01	0.99–1.00
EF	0.3	0.08	0.08–1.16	Vascular disease	1.67	0.12	0.88–3.18
LVEDD	1	0.88	0.98–1.03				
LVESD	1.01	0.29	0.99–1.04				
LVOT max	1	0.09	1.00–1.01				
Hypertension	1.46	0.02	1.06–1.99				
Diabetes	1.36	0.27	0.79–2.36				
Vascular disease	3.2	<0.001	1.68–6.07				
MWT	1.67	<0.001	1.29–2.16				
MWT^2^	0.99	<0.001	0.98–0.99				

AGE10, hazard ratio for 10‐year increments; age_af, interaction between age and AF; CI, confidence interval; FS, fractional shortening; HR, hazard ratio; LA5, hazard ratio for left atrial size for 5 mm increments; LVEDD, left ventricular end‐diastolic dimension; LVESD, left ventricular end‐systolic dimension; LVOT max: maximum LV outflow gradient; MWT, maximal wall thickness; TE, thrombo‐embolic; VKA, vitamin K antagonist.

MWT and MWT^2^ in the last two rows of the table adjust for MWT and its square term.

Age, AF, the interaction between age and AF, TE prior to first evaluation, NYHA class II, NYHA class III and IV, LA diameter, vascular disease, maximal LV wall thickness, and (maximal LV wall thickness)^2^ were included in the risk model. The estimates of the HRs and the corresponding CIs for the risk prediction model are shown in *Table* 3. There was no significant centre effect as part of a sensitivity analysis (shown in Supplementary material online, *Table S3*).

The risk of TE in 5 years for an individual HCM patient can be calculated from the following equation:

$$P_{TE\;\mathrm{at}\;5\;\text{years}}=1–\;0.9999874^{\exp\;\left(\text{prognostic index}\right)}$$

where the prognostic index = 0.030417476 × age (years) + 2.129977874 × af (yes = 1/no = 0) – 0.027069595 × age × af + 1.288557829 × TE prior (yes = 1/no = 0) + 0.224673046 × nyha class II (yes = 1/no = 0) + 0.728180341 × nyha class III/IV (yes = 1/no = 0) + 0.032251831 × la diam (mm) + 0.3735254 × mwt (mm) –0.008324216 × mwt2 (mm) + 0.512492795 × vascular disease (yes = 1/no = 0).

### Model validation

Bootstrapping showed a good calibration slope of 0.91 (95% CI 0.74–1.08). *Figure S1* in the Supplementary material online illustrates a good agreement between the observed and predicted risk at exploratory thresholds of thrombo‐embolic risk at 5 years. The C‐index was 0.75 (95% CI 0.70–0.80) and the D‐statistic was 1.30 (95% CI 1.05–1.56), indicating good discrimination.

### Comparison with conventional stroke prediction models

A total of 222 patients with complete data and AF were not treated with a VKA at baseline evaluation; of these, 61 (27.5%) had a CHA_2_DS_2_‐VASc score of 0 and 19 (8.6%) had a score between 4 and 6. No patient had a score of 7–9. *Table S4* of the Supplementary material online presents the prevalence of TE in patients according to CHA_2_DS_2_‐VASc score.

**Figure 1:**
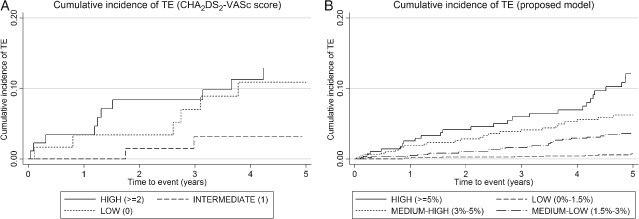
Kaplan–Meier failure estimates for cumulative incidence of thrombo‐embolism (TE). (A) The cumulative incidence of TE according to CHA_2_DS_2_‐VASc score groups (low = 0, 61 patients; intermediate = 1, 72 patients; high > 2, 89 patients). (B) The cumulative incidence of TE according to the 5‐year risk prediction model (low = 0–1.5%, 1643 patients; medium–low = 1.5–3%, 887 patients; medium–high = 3–5%, 494 patients; high >5%, 287 patients).

*Figure* 1 displays the cumulative incidence of TE according to CHA_2_DS_2_‐VASc score groups and according to the 5‐year risk prediction model.

### Relationship between anticoagulation and thrombo‐embolism risk in patients with atrial fibrillation

**Figure 2:**
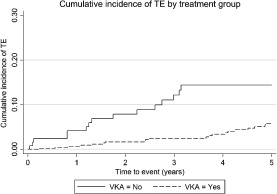
Kaplan–Meier failure estimates comparing thromb‐ oembolic events (TE) in vitamin K antagonist (VKA) and non‐VKA groups.

*Table S5* of the Supplementary material online presents an unadjusted exploratory analysis of the prevalence of TE over 10 years in patients with AF at first evaluation that did or did not receive a VKA during the follow‐up period prior to TE; 12.4% of those not receiving a VKA and 6.8% of patients of those who were receiving anticoagulation had a thrombo‐embolic event. This corresponds to a relative risk reduction of 54.8% (95% CI 0.31–0.97, *P* = 0.037) with VKA treatment. *Figure* 2 displays the Kaplan–Meier curves comparing VKA and non‐VKA groups. The ARR and NNT for patients in AF at an exploratory threshold of 4% risk of a thrombo‐embolic event over 5 years is 13% (95% CI 2.1–24%) and 7.7, respectively. These results should be interpreted with caution as the small numbers meant that a standard multivariable model adjusting for warfarin and the rest of the predictors was not appropriate. Instead we fitted a multivariable model as a sensitivity analysis using Lasso regression, a penalized regression method suitable for data sets with few events.^40^ In this fully adjusted analysis, warfarin maintained its protective effect (results not shown).

We also used propensity score analysis to explore further the indication for the effect of VKA on stroke. The propensity score for each patient was calculated as the predicted probability of receiving a VKA. It is clinical practice in the participating centres to consider therapy with VKA in patients with significantly enlarged LA diameter. Indeed LA was the strongest balancing factor in the propensity score model to identify treatment allocation variables, while older age, history of diabetes, and smaller fractional shortening were also associated with higher probability of receiving a VKA. Subsequently a Cox regression model for the time to TE adjusting for VKA and the propensity score^41^ showed that VKA maintained its protective effect (HR 0.41; 95% CI 0.22–0.76).

### Relationship of left atrial size to risk of thrombo‐embolic events

**Figure 3:**
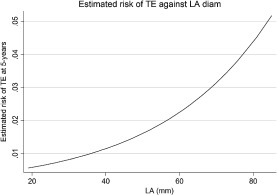
Relationship of left atrial (LA) size to risk of thrombo‐embolism (TE).

Figure 3 shows the relationship between LA size and 5‐year risk of TE. There appears to be a linear relationship up to ∼45–50 mm, at which point the risk of TE rises exponentially with increasing LA diameter. In the cohort of patients in SR at first evaluation with an LA diameter >50 mm, the prevalence of a thrombo‐embolic event was 4.7% (Supplementary material online, Table S6). Patients with an event in this group were older (55.8 years vs. 50.1 years) as compared with patients who did not experience an event. Patients in AF who did not develop a thrombo‐embolic event had a smaller LA diameter (50.0 mm vs. 52.3 mm) and were less symptomatic (NYHA III, IV) (19.6% vs. 44.7%) compared with patients with an event. The characteristics are displayed in the Supplementary material online, Table S1.

## Discussion

In this study we present the first validated model for TE prediction in a diverse population of adult patients with HCM. The study confirms the high risk of TE in patients with AF and the strong association between atrial size and thrombo‐embolic risk.^7^, ^8^, ^27^ It also demonstrates an association with several other patient characteristics including age, heart failure symptoms, and maximal LV wall thickness and, for the first time in this population, vascular disease.

### CHA_2_DS_2_‐VASc score in hypertrophic cardiomyopathy

Clinical guidelines recommend the CHA_2_DS_2_‐VASc score as a means of stratifying patients with non‐valvular AF for antithrombotic prophylaxis.^10^, ^12^, ^13^ We show that this score has a relatively low predictive accuracy in patients with HCM that is probably explained by the lower prevalence of vascular risk factors. These data support recent consensus guidelines from the European Society of Cardiology (ESC) that advise against the use of the CHA_2_DS_2_‐VASc score in patients with HCM.^1^

### Clinical outcome of anticoagulation in patients with atrial fibrillation

There are no prospective randomized trials of any therapy for AF in HCM including anticoagulation, and only a very small number of observational studies comparing VKAs with antiplatelet drugs.^7^, ^8^, ^42^, ^43^ In this study, an intention to treat analysis of VKA demonstrated a relative risk reduction for TE of 54.8% in anticoagulated patients who had AF at baseline evaluation, supporting current international guidelines for the use of VKAs in HCM.^1^, ^11^ The intention to treat analysis was felt to be the most appropriate way of analysing data gathered retrospectively over such a long period, although we acknowledge that this methodology may have led to the inclusion of patients who had discontinued VKA therapy or whose international normalized ratio (INR) was subtherapeutic at the time of a thrombo‐embolic event. Current guidelines recommend anticoagulation with a CHA_2_DS_2_‐VASc score of ≥1 in patients with non‐valvular AF, which corresponds to an adjusted stroke rate of 1.3% per year.^10^, ^12^, ^13^ The ARR and NNT for patients in AF at an exploratory threshold of 4% risk of a thrombo‐embolic event over 5 years is 13% (95% CI 2.1–24%) and 7.7, respectively.

### Implications for patients in sinus rhythm

The risk of TE in patients who are in SR has not been examined in detail previously. Risk factors for TE in this group included advanced age, heart failure symptoms, increased LA diameter, and vascular disease. While this study does not prove that patients in SR who have a high estimated risk of TE benefit from anticoagulation prior to the development of AF, it does support recent recommendations for frequent ambulatory ECG monitoring in patients with LA enlargement.^1^ Irrespective of atrial rhythm, clinicians should also be alert to conventional vascular risk factors and treat them appropriately.

### Limitations

The patient population in this study is large and diverse, but the model should only be used in patients with similar characteristics. It is not validated in paediatric patients (<16 years of age) or in patients with metabolic or syndromic disorders.

A prospective external validation in a different cohort of patients would be ideal.

Ethnicity may have influenced the findings. Data on this were not available in this cohort.

The model includes patients who are treated with a VKA according to current guidelines. Excluding these patients would exclude high risk patients and limit the statistical analysis.

## Conclusions

The study shows that the risk of TE in patients with HCM can be identified using a small number of simple clinical features. LA size, in particular, should be monitored closely, and the assessment and treatment of conventional vascular risk factors should be routine practice in older patients. Exploratory analyses show for the first time evidence for a reduction of TE with VKA treatment. The CHA_2_DS_2_‐VASc score does not appear to correlate well with the clinical outcome in patients with HCM and should not be used to assess TE risk in this population.

## Funding

This study was funded by the British Heart Foundation and by the National Institute for Health Research University College London Hospitals Biomedical Research Centre. It was also partially supported by the Red Investigación Cardiovascular from the Instituto de Salud Carlos III, Spanish Ministry of Health [grants RD12/0042/0049, RD12/0042/0066, and RD12/0042/0069].

**Conflict of interest:** O.P.G. received research support from the British Heart Foundation (FS/12/86/29841) and the National Institute for Health Research University College London Hospitals Biomedical Research Centre. All other authors declare no potential conflict of interest.

**Authors' contributions:** O.P.G. designed the study, collected and interpreted the data, carried out the descriptive statistical analysis, and wrote the article. P.M.E. designed the study, interpreted the data, and wrote the article. R.Z.O. was involved in study design, led the statistical aspects of the risk modelling, and wrote the article. M.P. carried out the statistical analysis. C.O'M., L.M., A.A., E.B., J.R.G., G.L., P.G.P., and C.R. collected and interpreted the data and critically reviewed the manuscript. W.J.M. was involved in the drafting of the article and revising it critically for important intellectual content. The additional investigators were involved in data collection and interpretation.

## Supporting information


**Figure S1** Agreement between observed and predicted risk of at exploratory thresholds of thrombo‐embolic risk at 5 years

**Table S1** Thrombo‐embolic events in patients with sinus rhythm and atrial fibrillation at baseline evaluation

**Table S2** Missing data per variable

**Table S3** Thrombo‐embolism risk prediction model and sensitivity analysis for centre effect

**Table S4** Prevalence of thrombo‐embolism according to CHA_2_DS_2_‐VASc score in hypertrophic cardiomyopathy patients with atrial fibrillation not treated with a vitamin K antagonist

**Table S5** Outcome of treatment with a vitamin K antagonist prior to an event in patients with atrial fibrillation at baseline evaluation with and without thrombo‐embolism

**Table S6** Thrombo‐embolic events in patients with sinus rhythm with left atrial size >45 and >50 at baseline evaluation
