# Supplementary material for: Prediction of thrombo‐embolic risk in patients with hypertrophic cardiomyopathy (HCM Risk‐CVA)
Source: Eur J Heart Fail. 2015 Jul 16;17(8):837–45. doi: 10.1002/ejhf.316 (PMC4737264; doi:10.1002/ejhf.316)
Supplement: Supplementary file 1 — Figure S1 Agreement between observed and predicted risk of at exploratory thresholds of thrombo‐embolic risk at 5 years [file EJHF-17-837-s001.doc]

**Supplementary figure 1:** Agreement between observed and predicted risk of at exploratory thresholds of thromboembolic risk at 5 years


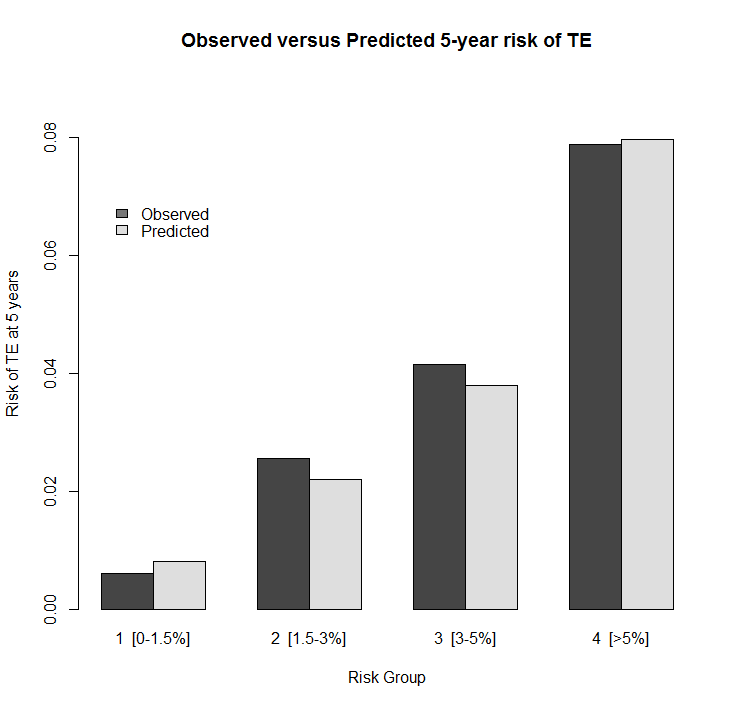
Group 1: 2464 patients, Group 2: 1274, Group 3: 696, Group 4: 387
