# Supplementary material for: Prediction of thrombo‐embolic risk in patients with hypertrophic cardiomyopathy (HCM Risk‐CVA)
Source: Eur J Heart Fail. 2015 Jul 16;17(8):837–45. doi: 10.1002/ejhf.316 (PMC4737264; doi:10.1002/ejhf.316)
Supplement: Supplementary file 2 — Table S1 Thrombo‐embolic events in patients with sinus rhythm and atrial fibrillation at baseline evaluation [file EJHF-17-837-s002.doc]

***Supplementary table 1:*** *Thromboembolic events in patients with sinus rhythm and AF at baseline evaluation*

|  |  | **SR** | **SR** | **AF** | **AF** |
| --- | --- | --- | --- | --- | --- |
|  | **characteristic** | **total** | **mean (SD)/n (%)** | **total** | **mean (SD)/n (%)** |
| **TE no** | **Age** | 4087 | 47.49 (16.29) | 552 | 57.87 (13.91) |
|  | **LA** | 3925 | 42.99 (7.15) | 531 | 50.03 (8.60) |
|  | **MWT** | 4047 | 19.43 (5.26) | 547 | 19.35 (4.51) |
|  | **FS** | 3719 | 0.41 (0.09) | 475 | 0.38 (0.11) |
|  | **LVOT max** | 3502 | 32.45 (41.48) | 516 | 27.70 (37.09) |
|  | **Female** | 4090 | 1436 (35.11) | 552 | 225 (40.76) |
|  | **Prior TE** | 4091 | 71 (17.36) | 552 | 0 |
|  | **VKA** | 4089 | 85 (2.08) | 552 | 323 (58.51) |
|  | **NYHA II** | 3921 | 1299 (33.13) | 525 | 219 (41.71) |
|  | **NYHA III,IV** | 3921 | 353 (9.00) | 525 | 103 (19.62) |
|  | **Hypertension** | 3995 | 1125 (28.16) | 540 | 227 (42.04) |
|  | **Diabetes** | 3363 | 214 (6.36) | 499 | 65 (13.03) |
|  | **FH SCD** | 3966 | 957 (24.13) | 539 | 111 (20.60) |
|  | **Vascular disease** | 2933 | 58 (1.98) | 499 | 21 (4.21) |
| **TE yes** | **Age** | 124 | 55.00 (15.48) | 48 | 57.62 (15.20) |
|  | **LA** | 119 | 46.00 (7.82) | 48 | 52.35 (8.04) |
|  | **MWT** | 121 | 20.38 (4.08) | 48 | 19.23 (4.27) |
|  | **FS** | 114 | 0.41 (0.09) | 46 | 0.38 (0.10) |
|  | **LVOT max** | 101 | 39.98 (41.33) | 44 | 22.66 (36.00) |
|  | **Female** | 124 | 53 (42.74) | 48 | 21 (43.75) |
|  | **Prior TE** | 124 | 9 (7.26) | 48 | 0 |
|  | **VKA** | 124 | 7 (5.64) | 48 | 26 (54.17) |
|  | **NYHA II** | 118 | 54 (45.76) | 47 | 11 (23.40) |
|  | **NYHA III,IV** | 118 | 17 (14.41) | 47 | 21 (44.68) |
|  | **Hypertension** | 123 | 37 (30.08) | 48 | 23 (47.92) |
|  | **Diabetes** | 108 | 9 (8.33) | 44 | 5 (11.36) |
|  | **FH SCD** | 120 | 32 (26.67) | 48 | 8 (16.67) |
|  | **Vascular disease** | 106 | 6 (5.66) | 44 | 4 (9.10) |

SD: Standard deviation, n: Number, LA: Left atrial size, MWT: Maximal wall thickness, FS: Fractional shortening, LVOT max: maximum LV outflow gradient, TE: thromboembolic event, AF: atrial fibrillation, VKA: Vitamin K antagonist, NYHA: New York Heart Association Functional classification, FH SCD: Family history of sudden cardiac death
