# Supplementary material for: Prediction of thrombo‐embolic risk in patients with hypertrophic cardiomyopathy (HCM Risk‐CVA)
Source: Eur J Heart Fail. 2015 Jul 16;17(8):837–45. doi: 10.1002/ejhf.316 (PMC4737264; doi:10.1002/ejhf.316)
Supplement: Supplementary file 3 — Table S2 Missing data per variable [file EJHF-17-837-s003.doc]

**Supplementary table 2:** Missing data per variable

| **predictor** | **n** | **Total** | **%** |
| --- | --- | --- | --- |
| **AF** | 6 | 4,821 | 0.12 |
| **VKA** | 3 | 4,821 | 0.06 |
| **Prior TE** | 0 | 4,821 | 0 |
| **sex** | 1 | 4,821 | 0.02 |
| **Age** | 4 | 4,821 | 0.08 |
| **NYHA class** | 206 | 4,821 | 4.27 |
| **MWT** | 53 | 4,821 | 1.1 |
| **LA** | 194 | 4,821 | 4.02 |
| **LVEDD** | 218 | 4,821 | 4.52 |
| **LVESD** | 452 | 4,821 | 9.38 |
| **LVOT max** | 653 | 4,821 | 13.54 |
| **FH SCD** | 142 | 4,821 | 2.95 |
| **Hypertension** | 109 | 4,821 | 2.26 |
| **Diabetes** | 801 | 4,821 | 16.61 |
| **Vascular disease** | 1,233 | 4,821 | 25.58 |

n: Number, AF: atrial fibrillation, VKA: Vitamin K antagonist, TE: thromboembolic event, NYHA: New York Heart Association Functional classification, MWT: Maximal wall thickness, LA: Left atrial size, LVEDD: Left ventricular end-diastolic dimension, LVESD: left ventricular end-systolic dimension, LVOT max: maximum LV outflow gradient, FH SCD: Family history of sudden cardiac death
