# Supplementary material for: Prediction of thrombo‐embolic risk in patients with hypertrophic cardiomyopathy (HCM Risk‐CVA)
Source: Eur J Heart Fail. 2015 Jul 16;17(8):837–45. doi: 10.1002/ejhf.316 (PMC4737264; doi:10.1002/ejhf.316)
Supplement: Supplementary file 4 — Table S3 Thrombo‐embolism risk prediction model and sensitivity analysis for centre effect [file EJHF-17-837-s004.doc]

**Supplementary table 3:** Thromboembolism risk prediction model and sensitivity analysis for centre effect

| **without centre** |  |  |  | **with centre** |  |  |
| --- | --- | --- | --- | --- | --- | --- |
| predictor | HR | p | CI | HR | p | CI |
| AGE | 1.03 | <0.001 | 1.02 - 1.04 | 1.030 | <0.001 | 1.02 - 1.04 |
| AF | 8.41 | <0.001 | 1.95 - 36.35 | 8.740 | <0.001 | 2.03 - 37.70 |
| age_af | 0.97 | 0.030 | 0.95 - 1.00 | 0.970 | 0.030 | 0.95 - 1.00 |
| prior TE | 3.63 | <0.001 | 1.81 - 7.29 | 3.600 | <0.001 | 1.78 - 7.28 |
| NYHA II | 1.25 | 0.210 | 0.88 - 1.78 | 1.230 | 0.250 | 0.86 - 1.75 |
| NYHA III, IV | 2.07 | <0.001 | 1.35 - 3.17 | 2.020 | <0.001 | 1.31 - 3.13 |
| LA | 1.03 | <0.001 | 1.01 - 1.05 | 1.030 | <0.001 | 1.01 - 1.05 |
| MWT | 1.45 | <0.001 | 1.12 - 1.88 | 1.460 | <0.001 | 1.13 - 1.89 |
| MWT^2^ | 0.99 | 0.010 | 0.99 - 1.00 | 0.990 | 0.010 | 0.99 - 1.00 |
| Vascular disease | 1.67 | 0.120 | 0.88 - 3.18 | 1.760 | 0.100 | 0.90 - 3.42 |
| Athens |  |  |  | 0.590 | 0.070 | 0.34 - 1.03 |
| Bologna |  |  |  | 0.880 | 0.660 | 0.50 - 1.55 |
| Corunña |  |  |  | 1.080 | 0.740 | 0.70 - 1.65 |
| Madrid |  |  |  | 0.660 | 0.380 | 0.26 - 1.67 |
| Murcia |  |  |  | 0.930 | 0.760 | 0.56 - 1.53 |
| Naples |  |  |  | 0.730 | 0.540 | 0.26 - 2.01 |

AF: atrial fibrillation, age_af: interaction between age and AF, TE: thromboembolic event, NYHA: New York Heart Association Functional classification, LA: Left atrial size, MWT: Maximal wall thickness, HR: Hazard ratio, p: p-value, CI: 95% confidence interval. Age: Hazard ratio for 10 year increments, LA: Hazard ratio for left atrial size for 5mm increments.
