# Supplementary material for: Prediction of thrombo‐embolic risk in patients with hypertrophic cardiomyopathy (HCM Risk‐CVA)
Source: Eur J Heart Fail. 2015 Jul 16;17(8):837–45. doi: 10.1002/ejhf.316 (PMC4737264; doi:10.1002/ejhf.316)
Supplement: Supplementary file 5 — Table S4 Prevalence of thrombo‐embolism according to CHA2DS2‐VASc score in hypertrophic cardiomyopathy patients with atrial fibrillation not treated with a vitamin K antagonist [file EJHF-17-837-s005.doc]

**Supplementary table 4:** Prevalence of thromboembolism according to CHA_2_DS_2_-VASc score in HCM patients with AF not treated with VKA.

| **CHA_2_DS_2_-VASc Score** | TE no, n (%) | TE yes, n (%) |
| --- | --- | --- |
| **0** | 55 (90.16%) | 6 (9.84%) |
| **1** | 69 (95.83%) | 3 (4.17%) |
| **2** | 37 (88.10%) | 5 (11.90%) |
| **3** | 26 (92.86%) | 2 (7.14%) |
| **4** | 11 (73.33%) | 4 (26.67%) |
| **5** | 2 (66.67%) | 1 (33.33%) |
| **6** | 1 (100%) | 0 (0%) |
| **Total** | 201 (90.54%) | 21 (9.46%) |

TE: Thromboembolic events, n: Number
