# Supplementary material for: Prediction of thrombo‐embolic risk in patients with hypertrophic cardiomyopathy (HCM Risk‐CVA)
Source: Eur J Heart Fail. 2015 Jul 16;17(8):837–45. doi: 10.1002/ejhf.316 (PMC4737264; doi:10.1002/ejhf.316)
Supplement: Supplementary file 6 — Table S5 Outcome of treatment with a vitamin K antagonist prior to an event in patients with atrial fibrillation at baseline evaluation with and without thrombo‐embolism [file EJHF-17-837-s006.doc]

**Supplementary Table 5:** Outcome of treatment with VKA prior to event in patients with AF at baseline evaluation with and without thromboembolism

| **AF** | **VKA** | **no TE** | **%** | **TE** | **%** |
| --- | --- | --- | --- | --- | --- |
|  | **no** | 113 | 87.60 | 16 | 12.40 |
|  | **yes** | 439 | 93.21 | 32 | 6.79 |
|  | **Total** | 552 | 92.00 | 48 | 8.00 |

AF: Atrial fibrillation, VKA: Vitamin K antagonist, TE: Thromboembolic events
