# Supplementary material for: Prediction of thrombo‐embolic risk in patients with hypertrophic cardiomyopathy (HCM Risk‐CVA)
Source: Eur J Heart Fail. 2015 Jul 16;17(8):837–45. doi: 10.1002/ejhf.316 (PMC4737264; doi:10.1002/ejhf.316)
Supplement: Supplementary file 7 — Table S6 Thrombo‐embolic events in patients with sinus rhythm with left atrial size >45 and >50 at baseline evaluation [file EJHF-17-837-s007.doc]

***Supplementary table 6:*** *Thromboembolic events in patients with sinus rhythm with LA>45 and sinus rhythm LA>50 at baseline evaluation*

|  |  | **SR, >45** | **SR, >45** | **SR, >50** | **SR, >50** |
| --- | --- | --- | --- | --- | --- |
|  | **variable** | **total** | **mean (SD)/n (%)** | **total** | **mean (SD)/n (%)** |
| **TE no** | **Age** | 1499 | 50.45 (14.94) | 728 | 50.15 (15.08) |
|  | **LA** | 1336 | 50.78 (4.40) | 565 | 54.80 (3.86) |
|  | **MWT** | 1478 | 20.72 (5.19) | 710 | 21.10 (5.37) |
|  | **FS** | 1291 | 0.41 (0.10) | 567 | 0.41 (0.11) |
|  | **LVOT max** | 1275 | 42.68 (46.60) | 608 | 45.12 (47.95) |
|  | **Female** | 1502 | 444 (29.56) | 731 | 193 (26.40) |
|  | **Prior TE** | 1502 | 34 (2.26) | 731 | 14 (1.92) |
|  | **VKA** | 1500 | 62 (4.13) | 729 | 43 (5.90) |
|  | **NYHA II** | 1434 | 526 (36.68) | 692 | 245 (35.40) |
|  | **NYHA III, IV** | 1434 | 192 (13.39) | 692 | 118 (17.05) |
|  | **Hypertension** | 1462 | 465 (31.81) | 711 | 227 (31.93) |
|  | **Diabetes** | 1223 | 102 (8.34) | 593 | 48 (8.09) |
|  | **FH SCD** | 1461 | 330 (22.59) | 708 | 153 (21.61) |
|  | **Vascular disease** | 1114 | 22 (1.97) | 543 | 9 (1.66) |
| **TE yes** | **Age** | 62 | 56.20 (12.35) | 34 | 55.75 (11.94) |
|  | **LA** | 57 | 52.12 (6.08) | 29 | 56.03 (6.27) |
|  | **MWT** | 59 | 20.92 (4.35) | 32 | 21.97 (4.98) |
|  | **FS** | 56 | 0.42 (0.09) | 30 | 0.41 (0.10) |
|  | **LVOT max** | 51 | 42.96 (41.55) | 29 | 41.76 (32.80) |
|  | **Female** | 62 | 24 (38.71) | 34 | 11 (32.35) |
|  | **Prior TE** | 62 | 6 (9.68) | 34 | 4 (11.76) |
|  | **VKA** | 62 | 6 (9.68) | 34 | 5 (14.71) |
|  | **NYHA II** | 59 | 28 (47.46) | 31 | 12 (38.71) |
|  | **NYHA III, IV** | 59 | 8 (13.56) | 31 | 7 (22.58) |
|  | **Hypertension** | 61 | 19 (31.15) | 34 | 11 (32.35) |
|  | **Diabetes** | 55 | 6 (10.01) | 27 | 2 (7.41) |
|  | **FH SCD** | 59 | 14 (23.73) | 32 | 9 (28.13) |
|  | **Vascular disease** | 55 | 5 (9.10) | 28 | 4 (14.29) |

SD: Standard deviation, n: Number, LA: Left atrial size, MWT: Maximal wall thickness, FS: Fractional shortening, LVOT max: maximum LV outflow gradient, TE: thromboembolic event, AF: atrial fibrillation, VKA: Vitamin K antagonist, NYHA: New York Heart Association Functional classification, FH SCD: Family history of sudden cardiac death
